# Prognosis and Outcome of Cervical Primary Extraosseous Intradural Extramedullary Ewing Sarcoma: A Systematic Review

**DOI:** 10.7759/cureus.26665

**Published:** 2022-07-08

**Authors:** César M Carballo Cuello, Orlando De Jesus, Aixa de Jesús Espinosa, Ricardo J Fernández-de Thomas, Gisela Murray, Emil A Pastrana

**Affiliations:** 1 Neurosurgery, University of Puerto Rico, Medical Sciences Campus, San Juan, PRI

**Keywords:** primitive neuroectodermal tumor, intradural, ewing sarcoma, extraosseous, extramedullary, cervical

## Abstract

Primary spinal extraosseous Ewing sarcoma (ES) is a rare mesenchymal tumor characterized by high malignancy, occurring in a few patients with ES. The occurrence of this tumor in the intradural extramedullary spinal region is infrequent. This systematic review examines primary extraosseous intradural extramedullary ES in the cervical region to provide specific outcomes and evaluate the role of adjuvant chemoradiation in overall prognosis. A systematic review was conducted to identify all cervical primary extraosseous intradural extramedullary ES reported in the literature. The search included the databases of PubMed, Google Scholar, Medline, Embase, and Scopus from inception to June 2021. Inclusion criteria include a reported death outcome or a minimum one-year follow-up.

Our search retrieved 21 articles that involved the cervical spine, but only 11 cases met the inclusion criteria. Of the nine patients who demonstrated disease progression, six experienced local failure, two had distant craniospinal axis failure, and one had systemic metastases. Five patients died of the disease at a median of 11 months after diagnosis. Our analysis showed a one-, two-, and five-year progression-free survival (PFS) of 36.4%, 36.4%, and 12.1%, respectively. The one-, two-, and five-year overall survival rates were 72.7%, 62.3%, and 46.8%, respectively. Three of the five (60%) patients who died received craniospinal radiotherapy. Of the six patients who survived, two received craniospinal radiotherapy (33%), and one received whole spine radiotherapy (17%). This review showed that patients with cervical primary extraosseous intradural extramedullary ES had poor progression-free survival and overall survival rates. The addition of adjunct craniospinal radiotherapy did not improve the prognosis of these patients.

## Introduction and background

Ewing sarcoma (ES) classically belonged to a family of histologically similar high-grade small round cell tumors that include osseous ES, extraosseous ES, peripheral primitive neuroectodermal tumor (pPNET), and Askin tumor (thoracopulmonary PNET) [1,2]. ES primarily occurs in children and adolescents and belongs to a subset of PNET. Despite their similarities in histologic features, ES differs from the other sarcomas in its clinical aggressiveness and molecular profile, which consists of a specific translocation involving FLI1 on chromosome 11q24 and breakpoint region 1 (EWSR1) on chromosome 22; most notably t(11;22)(q24;q12), producing the EWSR1-FLI1 fusion transcript protein [3,4]. Thus, based on its molecular profile, the 2020 World Health Organization (WHO) reclassified ES as a separate entity from three other subsets of round cell sarcomas [3].

While primary spinal extraosseous ES is a rare mesenchymal tumor characterized by high malignancy occurring in 9.8% of the patients with ES, spinal intradural extramedullary ES represents a much lower frequency [5]. Approximately, 20-24% of spinal intradural extramedullary ES occur within the cervical region [4,6]. The pathogenesis of developing an intradural ES tumor is unclear; however, studies have shown that expression of EWSR1-FLI1 fusion transcript up-regulates the expression of neural crest genes in bone marrow cells, fibroblasts, and other cell types, which may contribute to its development [7-10]. The prognosis of ES in the cervical spine may be worse than in other spinal regions in the thoracic or lumbosacral spine, given the greater risk for neurologic deficits and increased difficulty in gross total resection.

Previous systematic and literature reviews of spinal ES cases have discussed outcomes, combining all anatomical spinal regions into a unique group [4,6,11,12]. The outcomes of primary intradural extramedullary ES located in the cervical region have not been analyzed. This study aimed to determine and analyze the progression-free survival (PFS) and overall survival (OS) for cervical primary intradural extramedullary ES. In addition, the role of craniospinal radiotherapy on intradural extramedullary ES local disease control, distal neuroaxis disease control, and long-term survival was assessed.

## Review

Methods

Protocol

This systematic review of the literature was structured following the Preferred Reporting Items for Systematic Reviews and Meta-Analysis Statement (PRISMA-Statement) guidelines and protocol [13].

Eligibility Criteria, Information Sources, and Search Strategy

To identify all cervical primary extraosseous intradural extramedullary ES reported in the literature, we searched the electronic databases of PubMed, Google Scholar, Medline, Embase, and Scopus from inception to June 2021. The search strategy included the use of controlled vocabulary (MeSH terms) and the following keywords: cervical, spine, intradural, Ewing sarcoma, peripheral primitive neuroectodermal tumor, and extramedullary. These keywords were used in conjunction with the Boolean operators AND and OR. The search strategy was not changed between databases. If a published article was duplicated, the most detailed version was reviewed. Only studies written in English were considered for inclusion.

Selection Process

Two independent reviewers (CCC and ODJ) performed the initial screening of all studies based on article titles and abstracts to assess inclusion eligibility. Disagreement in the articles selected was resolved by consensus among both reviewers and the senior author (EP). Articles were included if they had a documented pathology-proven cervical primary intradural extramedullary ES. Eligibility for inclusion required that the study have at least a one-year follow-up or a death outcome.

Data Collection Process, Items, and Primary Endpoint

Two independent reviewers extracted all the data. Discrepancies were resolved by discussion between the reviewers. The following data were extracted from each study utilizing a standardized data extraction table: age, gender, degree of resection, adjuvant treatment, PFS, and OS. For this systematic review, the primary endpoint assessed from each study was the OS and local tumor control following surgery. Survival was determined from the time of diagnosis until death or the last recorded patient follow-up.

Data Analysis

For this review, descriptive statistics of gender, extent of resection, and adjuvant treatment were used. Statistical analysis for progression and survival was performed using Kaplan-Meier survival analysis using R statistical software (version 4.0.5, RStudio, Boston, MA).

Study Risk of Bias Assessment, Effect Measures, and Synthesis Methods

Each included study was independently assessed for risk of bias and methodological quality by two reviewers (C.C.C and O.D.J) using the ROBINS-I tool for non-randomized studies [14]. Where possible, the focus was on studies with the least potential for bias. The two observers critically appraised the articles independently. The two independent reviewers determined the level of evidence, sample size, data source/outcome assessors, and follow-up for each study. A third senior author reviewer (E.A.P) served as an arbitrator when there was disagreement between the primary reviewers. The quality assessment was based on the presence of an outcome for the patient. The overall quality of evidence was based on studies with the lowest risk of bias. However, all studies available included only case reports. The data extracted from the included articles were placed independently by both observers in a table for further analysis.

Results

Study Selection, Study Characteristics, and Results of Individual Studies

**Figure 1:**
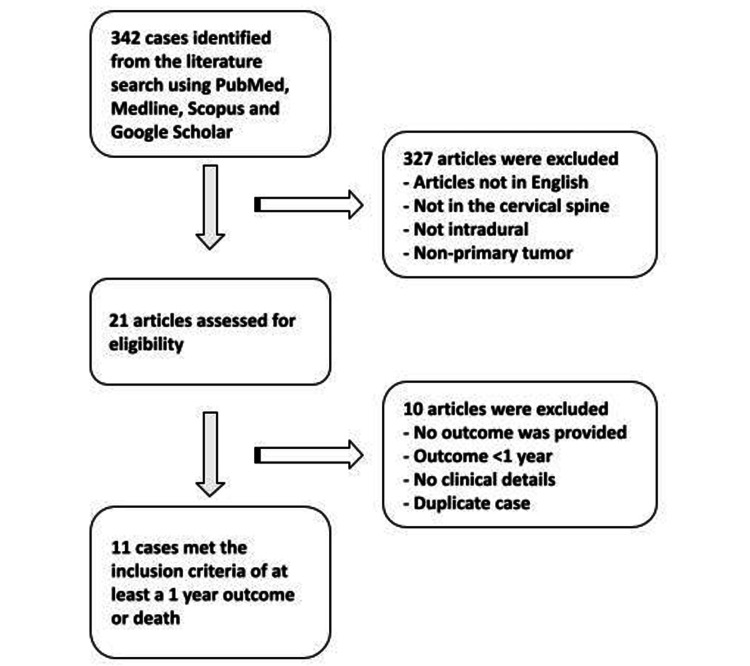
Flow diagram of studies in the review. Inclusion criteria required that the study documented a cervical primary extraosseous intradural extramedullary Ewing sarcoma with at least a one-year follow-up or a death outcome. Studies without clinical details were excluded.

The initial combined systematic literature search yielded 342 articles (Figure 1). Of these, 21 articles were assessed for eligibility as they included a cervical primary intradural extramedullary ES. Only 11 articles met the inclusion criteria and had complete clinical treatment information and at least a one-year follow-up or a death outcome (Table 1) [15-25].

**Table 1:** Descriptions of the patients with cervical spine intradural extramedullary Ewing sarcoma with outcomes of at least 12 months or a death event. ASCT, autologous stem cell transplant; C, chemotherapy; CS-R, craniospinal radiotherapy; DOD, died of disease; G, gender; TR, total resection, m, months; NED, no evidence of disease; R, radiotherapy; STR, subtotal resection.

Author	Age/G	Resection	R/C	CS-R	Outcome
Sevick et al. [15]	23/M	TR	R	Yes	Systemic metastases 30m, DOD 36m
Izycka-Swieszewska et al. [16]	26/M	STR	R	Yes	Progression, DOD 3m
Harimaya et al. [17]	30/F	STR	R/C	No	Local recurrence 4m, DOD 14m
Yan et al. [18]	10/M	TR	None	No	Leptomeningeal spread, progression, DOD 1m
Tan et al. [19]	34/F	STR	R	Yes	Local recurrence 9m, DOD 11m
Kim and Shin [20]	32/F	STR	R/C	No	NED, alive 12m
Alexander et al. [21]	45/M	STR	R	Yes	Local recurrence 1m, alive 25m
Gong et al. [22]	39/F	TR	R/C	No	Distant recurrence at 36m, then C, alive 36m
Chihak et al. [23]	25/M	STR	R/C	Yes	Progression 3 weeks post-op, reoperation, then R/C, NED, alive 62m
Margol et al. [24]	15/F	TR	R/C	No	NED, alive 84m
Bostelmann et al. [25]	29/M	TR	R/C	Whole spine	Multiple recurrences local 1m, distal 6m, then R/C, ASCT, NED, alive 18m

Outcomes

**Figure 2:**
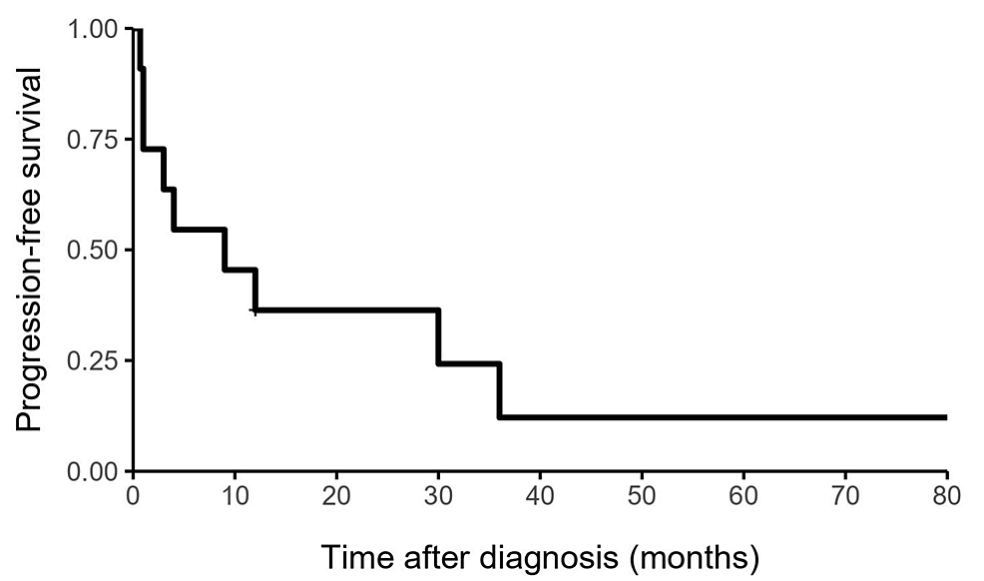
Kaplan-Meier curve for progression-free survival.

**Figure 3:**
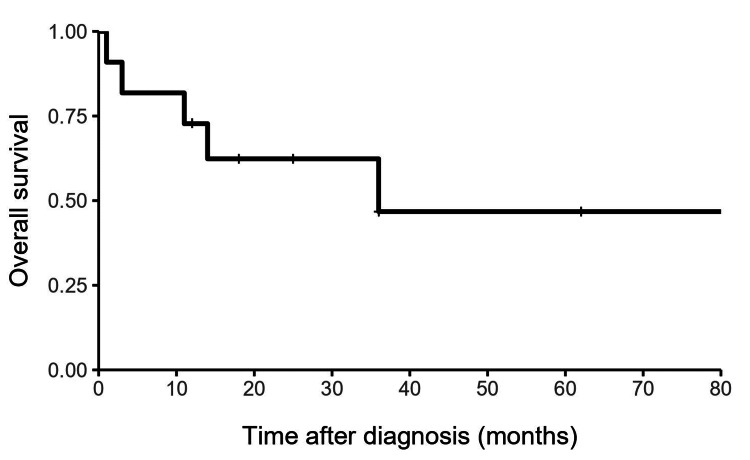
Kaplan-Meier curve for overall survival.

Analysis of these 11 cases showed six male patients and five females. All patients underwent surgical resection; five (45%) had a gross total resection, and six (55%) had a subtotal resection. Three of the six surviving patients had a complete resection, and three had a subtotal resection. Two of the five deceased patients had a complete resection, and three had a subtotal resection. All five patients died from disease progression at a median of 11 months after diagnosis. Nine patients (82%) developed disease progression following surgery, with eight of whom (89%) developed progression within the central nervous system (CNS), while one patient (11%) developed recurrence outside of the CNS. Among these nine patients, six (67%) experienced primary site failure, two (22%) had distant craniospinal axis failure, and one (11%) had systemic metastases. Five of the eight patients (63%) with disease progression within the CNS had a subtotal resection. Our review of cervical primary intradural extramedullary ES showed a one-year PFS of 36.4%, a two-year PFS of 36.4%, and a five-year PFS of 12.1% (Figure 2). The OS was 72.7% at one year, 62.3% at two years, and 46.8% at five years (Figure 3).

Radiotherapy

Six patients (55%) were treated with a combination of adjuvant chemotherapy and radiotherapy, while four (36%) were treated only with radiotherapy. One patient (9%) did not receive adjuvant treatment and died from leptomeningeal spread 30 days after his diagnosis. One patient (9%) received complete spinal radiotherapy, five (45%) received craniospinal radiotherapy, and four (36%) received only local radiotherapy. The dose of local radiotherapy ranged from 30 to 54 Gy; however, the dose for craniospinal radiotherapy was uniformly 36 Gy. Three of the five (60%) patients who died received craniospinal radiotherapy. Of the six patients who survived, two (33%) received craniospinal radiotherapy, and one (17%) received complete spinal radiotherapy.

Chemotherapy

Two (18%) patients had distant craniospinal axis failure with recurrence to another spinal region. One received neoadjuvant chemotherapy and local radiotherapy after the initial diagnosis and was treated with subtotal resection followed by additional chemotherapy when the distant spinal recurrence was identified. The other patient only received neoadjuvant chemotherapy after the initial diagnosis and was treated with debulking surgery and adjuvant craniospinal radiotherapy. Additional chemotherapy was given when the distant spinal recurrence was identified. This patient was the only one who received an autologous stem cell transplant and was alive 18 months after treatment. Only one out of the five deceased patients (20%) received adjuvant chemotherapy, while five of the six patients (83%) who survived received chemotherapy and radiotherapy.

Risk of Bias in Studies, Reporting Biases, Certainty of Evidence

We found that the risk of bias was not serious within all included studies. Only studies showing a death outcome or follow-up of at least 12 months were included. The quality of evidence was determined for all studies using the GRADE system. All the evidence for the benefits of craniospinal radiotherapy and survival comes from single-case reports. The quality of evidence was low due to all studies being individual case reports.

Discussion

Outcomes

Primary intradural extramedullary ES most commonly occurs in the lumbar region, followed by the cervical region [4]. In our review of the cervical spinal cases, patients presented at a median age of 29 years, with a 55% male predominance. This is consistent with the presentation in all spinal regions, where the median age of presentation is 32 years, with a 62% male predominance [4].

The outcomes of primary intradural extramedullary ES specifically located in the cervical region have not been previously analyzed. Previous reviews of spinal ES cases have discussed outcomes, combining all anatomical spinal regions into a unique group. Chihak et al. showed that primary spinal intradural extramedullary ES had a poor prognosis with a median recurrence time of 18 months and a 58% two-year event-free (recurrence or death) outcome despite multimodality therapy [23]. Forty-one percent of these patients experienced distant craniospinal axis failure [23]. Similar studies have shown a median PFS of 12 months and a median overall OS of 14 months [6]. Stratification of all reported intradural extramedullary ES revealed that the 1-, 2-, 3-, and 5-year PFS rates were 75.0%, 56.3%, 37.5%, and 18.8%, and OS rates were 89.5%, 80.5%, 80.5%, and 43.0%, respectively [4]. Similar conclusions were reached by Izubuchi et al., demonstrating that the one-, two-, and five-year PFS rates for all intradural extramedullary ES were 61.0%, 52.3%, and 10.9%, and the one-year and five-year OS rates were 79.8% and 26.6%, respectively [12]. Primary intradural extramedullary ES is more aggressive and has a poorer prognosis than its osseous counterpart [12].

Multiple hypotheses for the poor prognosis have been proposed. First, patients commonly receive intralesional subtotal resections, which increases the probability of microscopic tumor dissemination via the cerebrospinal fluid [17,26]. Sixty-three percent of the patients in our study whose disease progressed within the CNS had an intralesional subtotal resection. Second, the surgical resections might occur more emergently due to neurologic compromise, thus precluding the role of neoadjuvant chemoradiation [16,23]. Half of the patients who demonstrated CNS progression following an intralesional subtotal resection were noted to have the tumor densely adherent to the cord and nerve roots [19-21]. And lastly, oncologic resections with negative margins could not be achieved. Total "en bloc" resection and adjuvant radiotherapy were independent prognostic factors that significantly improved PFS and OS in patients with primary osseous spinal ES [27]. Sciubba et al. recommended "en bloc" surgical resection when feasible as it provided improved local control; however, it did not improve OS for osseous spinal ES [26]. These findings from osseous ES were extrapolated to intradural extramedullary ES with the recommendation that complete resection, if possible, be performed together with adjuvant chemotherapy and radiotherapy [6]. In our review, the extent of resection did not improve the outcome of patients with tumors in the cervical spine, thus emphasizing a significant difference between osseous ES and intradural intramedullary ES.

Yan et al. showed that the tumor location and spinal cord level involved were independent factors in the prognosis of spinal ES patients, with the worst prognosis in patients with intradural and higher spinal cord level involvement [18]. In our review of cervical cases, the one-, two-, and five-year PFS rates were 36.4%, 36.4%, and 12.1%, and the one-, two-, and five-year OS rates were 72.7%, 62.3%, and 46.8%, respectively. Based on these results, patients with ES in the cervical spinal region appear to have worse initial one- and two-year PFS and OS rates than previously reported outcomes in patients with tumors in any spinal region.

Craniospinal Radiotherapy

Similar to the standard treatment for central PNET, the addition of craniospinal axis radiotherapy has been recommended for spinal ES as it can potentially enhance survival [16,21]. Chihak et al. describe that craniospinal radiotherapy was critical for preventing distant recurrence and observed no evidence of recurrence in patients treated with craniospinal radiotherapy compared to patients who only had local radiotherapy to the primary disease site [23]. Izubuchi et al. reported a patient with a thoracolumbar ES with skip lesions in other spine regions but none in the brain [12]. The patient underwent surgery, chemotherapy, and whole-spine radiotherapy without brain irradiation but eventually developed brain metastases and died. Based on the outcome, the authors thought that craniospinal radiotherapy was more effective than whole spine radiotherapy for disease control, therefore recommending it in addition to surgery and chemotherapy. Our review showed that 83% of the patients who survived received a combination of chemotherapy and radiotherapy. Sixty-seven percent of the patients who received craniospinal radiotherapy or whole spine radiotherapy developed disease progression with local and systemic recurrences; however, none developed brain metastases. Both patients who developed a distant craniospinal axis recurrence did not receive craniospinal radiotherapy before the distant spinal recurrence was identified. In these two patients, craniospinal radiotherapy may have been effective in preventing craniospinal axis failure. Sixty percent of the patients who died received craniospinal radiotherapy. Fifty percent of the patients who survived received craniospinal radiotherapy or whole spine radiotherapy. Our review found no conclusive evidence to suggest that craniospinal radiotherapy improves the prognosis or the OS of patients with cervical primary extraosseous intradural extramedullary ES.

Chemotherapy

Standard chemotherapy protocols include combinations of vincristine, doxorubicin, cyclophosphamide, ifosfamide, and etoposide [23,27]. Harimaya et al. recommended multi-agent chemotherapy combined with "en bloc" resection and radiotherapy as the preferred treatment for patients with ES around the spinal column. In our review, most patients treated in the last decade were alive at the time of the report [17]. The advent of newer chemotherapy protocols and radiotherapy may have improved the recurrence and survival rates in patients with cervical primary extraosseous intradural extramedullary ES. Neoadjuvant chemotherapy is strongly recommended based on moderate evidence for managing ES of the spine as it significantly improves local control and long-term survival [26]. On multivariable analysis, Chen et al. found that adjuvant chemotherapy was not an independent prognostic factor for PFS and OS. In the future, a role for genomic-guided oncologic therapy may improve the survival of these patients [27].

Limitations

The first limitation of this study is the limited number of cervical intradural extramedullary ES cases reported in the literature. Several included patients had significantly short follow-ups, thus introducing selection bias in our Kaplan-Meier OS curves. A small number of cases were excluded from analysis due to limited follow-up or because the authors did not report sufficient clinical data. There was a wide variation in the timing and dosing of adjuvant and neoadjuvant chemotherapy regimens. Improvements in surgery, such as microdissection techniques and intraoperative neuro-monitoring, may have led to different outcomes in some of the cases included in the review. Lastly, some of the previously published cases were reported as ES/pPNET or pPNET and may have had different pathogenesis than the new classification of ES [3].

## Conclusions

This systematic review demonstrated that most patients with intradural Ewing sarcoma tumors in the cervical spine underwent a subtotal resection because of the difficulty of achieving a gross total resection due to the proximity of vital structures. The patients with cervical primary intradural extramedullary ES had poor PFS and OS rates. The addition of craniospinal radiotherapy did not improve the prognosis in these patients.
